# Association between systolic blood pressure parameters and unexplained early neurological deterioration (UnND) in acute ischemic stroke patients treated with mechanical thrombectomy

**DOI:** 10.1177/17562864221093524

**Published:** 2022-06-16

**Authors:** Aleksandras Vilionskis, Virginija Gaigalaite, Lukas Salasevicius, Dalius Jatuzis

**Affiliations:** Institute of Clinical Medicine, Vilnius University, Vilnius, Lithuania; Institute of Clinical Medicine, Vilnius University, Siltnamiu 29, Vilnius 01513, Lithuania; Institute of Clinical Medicine, Vilnius University, Vilnius, Lithuania; Institute of Clinical Medicine, Vilnius University, Vilnius, Lithuania

**Keywords:** blood pressure, mechanical thrombectomy, neurological deterioration, stroke

## Abstract

**Background:**

Neurological deterioration (ND) after mechanical thrombectomy (MT) of acute ischemic stroke (AIS) in anterior circulation is an important complication associated with a poor outcome. Moreover, evident causes of ND may remain unexplained (UnND).

**Objective:**

We sought to evaluate the association of the systolic blood pressure (SBP) parameters before MT, during MT, and during a 24-h period after MT with UnND.

**Methods:**

We analyzed 382 MT-treated AIS patients in two stroke centers from 2017 to 2019. The patients with unsuccessful recanalization and/or with symptomatic intracerebral hemorrhage after MT were excluded. Multivariate logistic regression analysis was used to identify the SBP parameters that predict UnND.

**Results:**

There were 5.9% patients with UnND within 24 h after MT among patients with successful recanalization what comprises 4.9% of all patients who had undergone MT. SBP > 180 mmHg on admission (odds ratio (OR): 4, 95% confidence interval (CI): 1.6–10, *p* = 0.004) and a drop of SBP below100 mmHg during MT (OR: 4.7, 95% CI: 1.3–17, *p* = 0.019) were associated with UnND occurrence within 7 days without a significant association with UnND within 24 h. UnND within 7 days was predicted by the episodes of SBP exceeding the level of SBP observed before the groin puncture and occurring over the first 2 h following recanalization (OR: 5, 95% CI: 1.3–19, *p* = 0.021), an increase of SBP of more than 20% within 2–24 h after MT (OR: 3.4, 95% CI: 1.1–10, *p* = 0.035), and a drop of SBP below 100 mmHg after MT (OR: 3.2, 95% CI: 1.1–9, *p* = 0.039).

**Conclusion:**

The association between the SBP parameters and UnND depends on the treatment period and the time of UnND occurrence. The J/U resembling relationship between SBP and UnEND was established during a 24-h period after MT.

## Introduction

Mechanical thrombectomy (MT) alone or combined with intravenous tPA (tissue-type plasminogen activator) is one of the most effective treatments for acute ischemic stroke (AIS) in patients with large-vessel occlusion of the anterior circulation. Despite successful recanalization *via* MT, some patients develop early neurological deterioration (ND). An increase in ⩾4 points of the National Institutes of Health Stroke Scale (NIHSS) between pretreatment and Day 1 is commonly considered as an early ND,^1–4^ though for minor stroke patients an increase in ⩾2 points of the NIHSS is considered as ND.^1–5^ Several studies demonstrated that early or subacute ND is a strong predictor of poor stroke outcomes.^6^ Apart from obvious causes such as lack of recanalization, parenchymal hemorrhage, malignant edema, or procedural complications, ND might remain unexplained in over half of cases (UnND).^2–4^ Although hemodynamic factors are suspected as important mechanisms of ND, this has not been directly examined to date. Therefore, there are currently no management guidelines for UnND.

Several studies^5,7–12^ demonstrated an association between different blood pressure (BP) parameters in periprocedural period and a clinical outcome or hemorrhagic complications. However, due to heterogeneity of studies, it is still unknown whether steady parameters (different BP thresholds) or dynamic parameters (BP variability, which takes into account various BP variations) before, during, and after MT have the same impact on prognosis. In addition, the type of relationship (J/U shaped or other) between systolic blood pressure (SBP) and the outcome is a matter of discussion. Therefore, there is no clear consensus for the optimal BP control before, during, and after MT.^13,14^

The association between BP parameters in periprocedural period and UnND has been scarcely studied; therefore, their impact on UnND at different treatment phases is unclear. Girot *et al.*^2^ noted that the higher baseline SBP was associated with UnND within 24 h, despite the exclusion of patients with early symptomatic intracranial hemorrhage (sICH). Although blood pressure variability (BPV) during a 24-h period after MT was associated with poor clinical outcomes and is most likely to be apparent in patients who have been successfully recanalized,^5^ the association of BPV and ND is less clear, and the results are controversial.^15,16^

We aimed to investigate the association of various dynamic and steady SBP parameters with UnND in patients with successful recanalization and without hemorrhagic transformations in different treatment phases.

## Material and methods

This retrospective study was focused on patients aged ⩾18 years with AIS in anterior circulation who had undergone MT alone or combined with intravenous tPA at the two stroke centers of Lithuania from 2017 through 2019. All patients were treated according to the National guidelines for management of acute stroke patients. Patients with an absence of successful recanalization (defined as a final modified thrombolysis in Cerebral Infarction grade of 2b–3), sICH (as defined by the European Cooperative Acute Stroke Study Classification) after MT, or procedural complications (embolization of a new territory, perforation, or intracranial dissection) were excluded from the study. Hemorrhagic complications, infarction lesion were assessed by non-contrast cranial computed tomography (CT) scans performed within 24 h after MT and, in addition, any time was indicated by clinical manifestations.

## Baseline clinical information and BP parameters

The records of the baseline and treatment characteristics of the included patients were as follows: age, sex, comorbidities (hypertension, diabetes, heart failure, atrial fibrillation, and history of stroke), baseline blood glucose level, the presence of intravenous thrombolysis before MT, the time from the onset of a stroke to recanalization, baseline NIHSS score, and the type of anesthesia.

The SBP values were analyzed using various parameters of SBP records. For each individual, the following SBP parameters were measured: SBP on admission, SBP before groin puncture, the mean, maximum, minimum values for the SBP during MT and after MT within 24 h. The following parameters of the SBP variability were calculated: standard deviation of the mean (SD), coefficient of variation (CV) = SD/mean × 100). The magnitude of the SBP increase after MT with respect to the level of SBP observed before groin puncture were calculated at the following time points – immediately after recanalization, within 2, 4, 12, and within 24 h after MT. In addition, the maximal increase and the maximal decrease of SBP during MT in comparison with the level of SBP observed before groin puncture was calculated. The magnitude of SBP changes were expressed as a percentage.

### Outcome assessments

Early neurological improvement was defined as a decrease of NIHSS score by ⩾4 points in comparison with NIHSS on admission or NIHSS score = 0 or 1 within 24 h. ND within 24 h was defined as an increase of NIHSS score by ⩾4 points compared to the NIHSS on admission or death within 24 h. UnND was defined as ND not related to symptomatic intracranial hemorrhage (sICH) (as defined by the European Cooperative Acute Stroke Study classification) within 24 h, the absence of good recanalization (defined as a final modified thrombolysis in cerebral infarction grade of 2b–3), malignant edema and procedural complications (embolization of a new territory, perforation, or intracranial dissection), early recurrent ischemic stroke in a different arterial territory. UnND within 7 days was defined in a similar way with the measurement of NIHSS changes within 7 days. Apart from the above-mentioned causes, patients with early systemic medical complications (e.g. myocardial infarction, infection, and pulmonary embolism) were excluded from UnND group.

#### Statistical analysis

Descriptive **c**ontinuous variables were reported as mean ± SD or median (interquartile range) as appropriate. Categorical variables were reported as proportions. Between groups, comparisons for continuous/ordinal variables were made with Student *t*-test, Mann–Whitney *U*-test, or analysis of variance (ANOVA), as appropriate. Categorical variables were compared by χ^2^ test or Fisher exact test as appropriate. Significance was set at *p* < 0.05, and all *p* values were based on two-tailed tests.

The univariate logistic analysis was performed to determine an association between risk factors, and UnND within 24 h and within 7 days. The choice of potential confounders for multivariate models was primarily based on a review of previous studies.^5,7,8,10–19^ All covariates and BP parameters with an unadjusted *p* ⩽ 0.1 were included in an initial multivariate model. The analysis of excessive collinearity diagnostics was performed, including computation of pairwise correlation coefficients to assess the interdependence of causal variables, as well as measures of multicollinearity. The models’ goodness of fit was assessed by the Hosmer–Lemeshow test. Statistical analyses were performed using SPSS version 24.0.

##### Ethics statement

This study was approved by the Lithuanian Bioethics Committee (No. L-14-03/4). In accordance with the legal acts of the Republic of Lithuania, the informed patient consent was not required because only anonymized data collected retrospectively were used in this study.

## Results

A total of 382 patients with successful recanalization (TICI scores of 2b or 3) and without sICH after MT were analyzed. UnND within 24 h comprises 4.9% of all patients who underwent MT and 5.9% of all patients with successful recanalization.

The baseline characteristics of the study patients are listed in Table 1. Most patients (64.4%) received IV-tPA treatment prior to the MT procedure. The median SPB at admission was 158 mmHg, interquartile range 140–178 mmHg.

**Table 1.** Baseline characteristics of the study patients.

	Number of patients (percent), *N* = 382
Male, *n* (%)	152 (39.8)
Age in years, median (IQR)	74 (67–80)
Atrial fibrillation, *n* (%)	210 (55)
Diabetes mellitus, *n* (%)	50 (13.1)
Hypertension, *n* (%)	308 (80.6)
Congestive heart failure	70 (18.3)
Previous stroke, *n* (%)	42 (11)
TACS, *n* (%)	160 (41.9)
Baseline NIHSS score, median (IQR)	17 (13–20)
Previous use of IV thrombolysis, *n* (%)	246 (64.4)
General anesthesia during MT, *n* (%)	166 (43.5)
Time from the onset to recanalization (min), median (IQR)	248 (204–305)
SBP at admission mmHg, median, IQR	158 (140–178)

IQR, interquartile range; IV, intravenous; MT, mechanical thrombectomy; NIHSS, National Institutes of Health Stroke Scale; SBP, systolic blood pressure; TACS, total anterior circulation stroke.

The main difference between the baseline characteristics among the groups without UnND, with UnND and a group with an early neurological improvement within 24 h are detailed in Supplemental Table 1. Hypertension was significantly more frequent in UnND group while other characteristics did not differ between groups.

There was no association between SBP in terms of lowest SBP, highest SBP, mean of SBP among patients with UnND, and without UnND (or early neurological improvement) within 24 h in preinterventional, prerecanalizational, and posrecanalizational phases (Supplemental Table 2). Meanwhile, the difference between the highest and the lowest SBP during MT was more pronounced in the group with UnND in comparison with the group of early neurological improvement, although the significance was marginal (*p* = 0.05).

The univariate logistic regression model (Table 2) shows that the instances of SBP increase by 10% during the procedure, the episodes of SBP exceeding the level of SBP observed before groin puncture and occurring within a 2-h period after recanalization and a fall of SBP below 100 mmHg during a 2- to 24-h period after MT were associated with UnND within 24 h in patients with successful recanalization and without sICH after MT. The multivariate analysis model demonstrates that the episodes of SBP exceeding the level of SBP before groin puncture and occurring within a 2-h period after recanalization and a fall of SBP below 100 mmHg within 2–24 h after MT remained significantly associated with a higher likelihood of UnND within 24 h (Table 2). Owing to multicollinearity, hypertension was not included in the model.

**Table 2.** Logistic regression analysis of predictors of UnND within 24 h in patients with successful recanalization and no procedural and hemorrhage complications (*N* = 382).

	Univariate analysis		Multivariate analysis	
	Odds ratio, 95% CI	*p* value	Odds ratio, 95% CI	*p* value
Male	0.48 (0.2–1.2)	0.13		
Age per 10-year increase	0.9 (0.7–1.3)	0.7		
NIHSS score per 5 points increase	1.2 (0.8–1.6)	0.4		
Atrial fibrillation	1.2 (0.5–2.6)	0.7		
Diabetes mellitus	1.3 (0.4–4)	0.5		
Congestive heart failure	0.4 (0.1–1.6)	0.2		
Previous stroke	0.7 (0.2–3.2)	0.7		
Glucose level at admission > 6 mmol/l per 1 mmol/l increase	0.8 (0.3–2.4)	0.7		
General anesthesia	1.3 (0.6–3)	0.5		
IV thrombolysis before MT	1.7 (0.7–4.4)	0.2		
Time from onset to recanalization per 30-min increase	0.9 (0.8–1.2)	0.8		
SBP at admission > 180 mmHg	1.1 (0.4–3.5)	0.8		
SBP before groin puncture				
• SBP per 10 mmHg increase	0.82 (0.6–1.1)	0.26		
• SBP < 120 mmHg	2.4 (0.8–7,5)	0.13		
• SBP > 160 mmHg	0.7 (0.3–1.7)	0.47		
SBP changes during MT				
• SBP decrease > 20%	1.5 (0.3–1.9)	0.5		
• SBP per 10% increase	2 (1.2–3.5)	0.011	1.6 (0.8–3.5)	0.1
• Maximal SBP > 160 mmHg	1.16 (0.5–2.7)	0.7		
• Maximal SBP > 180 mmHg	0.8 (0.2–3.5)	0.7		
• Minimal SBP < 100 mmHg	1.6 (0.6–4.1)	0.3		
• SD of SBP per 1-unit increase	1.01 (0.97–1.05)	0.5		
• CV of SBP per 1-unit increase	2.9 (1–414)	0.6		
Episodes of SBP exceeding the level of SBP observed before groin puncture and occurring after MT				
• Within a 2-h period after recanalization	2.2 (0.9–5)	0.049	3.3 (1.4–7)	0.01
• Immediately after recanalization	3.4 (1.5–8)	0.005		
• Within 2–24 h after MT	1.5 (0.6–3.7)	0.35		
SBP changes within 24 h after MT				
• Increase of SBP in comparison with SBP after recanalization:				
• Increase of SBP > 10%	1.02 (0.6–1.6)	0.9		
• Increase of SBP > 20%	1.2 (0.6–2.7)	0.6		
• Decrease of SBP in comparison with SBP at the end of MT > 20%	1.5 (0.5–4.6)	0.4		
• Instances of SBP > 160 mmHg	1.3 (0.6–2.9)	0.5		
• Instances of SBP > 180 mmHg	0.6 (0.2–2.8)	0.5		
• Instances of SBP fall below 100 mmHg within 24 h after MT	1.4 (0.5–4.5)	0.2		
• Instances of SBP fall below 100 mmHg within 2–24 h after MT	3.4 (1.1–10)	0.04	2.1 (1.1–3.9)	0.014
• SD of SBP after MT per 1-unit increase	1 (0.95–1.06)	0.9		
• CV of SBP after MT per 1-unit increase	1.003 (0.99–1.017)	0.62		

CI, confidence interval; CV, coefficient of variation; IQR, interquartile range; IV, intravenous; MT, mechanical thrombectomy; NIHSS, National Institutes of Health Stroke Scale; SBP, systolic blood pressure; SD, standard deviation.

To reduce the influence of patients with a stable course after MT, the additional analysis of only two marginal groups with early neurological improvement and UnND within 24 h was made. The results were similar to the above-mentioned with a higher risk for females, as shown in Supplemental Table 3.

Only 2 of 24 patients with UnND observed within 24 h showed an improvement within a 7-day period. Both patients were less than 65 years, with SBP on admission less than 180 mmHg. SBP variation during procedure was from 125 to 130 mmHg and baseline NIHSS 20 and 24.

There were 34 patients with UnND on the 7th day. It comprises 6.9% of all patients who underwent MT and 8.3% of all patients with successful recanalization. Nine out of those patients had early neurological improvement within 24 h. Other baseline and hemodynamic characteristic are detailed in Supplemental Tables 4 and 5.

The results about the prediction of UnND within 7 days are presented in Table 3. The univariate analysis indicated that high SBP on admission (>180 mmHg), a fall of SBP below 100 mmHg during MT, an increase of SBP by 10% during MT procedure, episodes of SBP exceeding the level of SBP observed before groin puncture and occurring within a 2-h period after recanalization, an increase of SBP by more than 20% within 24 h after MT in comparison with SBP observed after MT, a fall of SBP below 100 mmHg within 2–24 h after recanalization were significantly associated with UnND within a 7-day period. However, instances of SBP > 160 mmHg or SBP > 180 mmHg during MT or within 24 h after MT did not demonstrate statistically significant effects on the UnND. The multivariate analysis showed that high SBP on admission (>180 mmHg), the episodes of SBP drop during MT below 100 mmHg, episodes of SBP exceeding the level of SBP observed before groin puncture and occurring within a 2-h period after recanalization, an increase of SBP by more than 20% within 24 h after MT, the episodes of SBP drop below 100 mmHg within 2–24 h after MT remained significantly associated with UnND within a period of 7 days. In agreement with our results, the J/U-resembling relationship between SBP and UnND was found only during a 24-h period after MT.

**Table 3.** Logistic regression analysis of independent predictors of UnND occurrence within a 7-day period in patients with successful recanalization and no procedural and hemorrhage complications (*n* = 382).

	Univariate analysis		Multivariate analysis	
	Odds ratio, 95% CI	*p* value	Odds ratio, 95% CI	*p* value
Male	1.07 (0.5–2.1)	0.8		
Age per 10-year increase	1.2 (0.8–1.6)	0.3		
Atrial fibrillation	1.2 (0.6–2.3)	0.67		
Diabetes mellitus	1.7 (0.6–4.3)	0.29		
Congestive heart failure	1.7 (0.8–3.8)	0.18		
Previous stroke	1.1 (0.4–3.3)	0.8		
Glucose level > 6 mmol/l per 1 mmol/l increase	0.95 (0.6–1.5)	0.8		
NIHSS score per 5 points increase	1.2 (0.8–1.8)	0.42		
IV thrombolysis before MT	1.9 (0.9–4.4)	0.11		
General anesthesia	1.5 (0.7–2.9)	0.3		
Time from onset to recanalization per 30-min increase	0.98 (0.8–1.2)	0.8		
SBP on admission > 180 mmHg	3.7 (1.7–8)	0.001	4 (1.6–10)	0.004
SBP before groin puncture				
• SBP per 10 mmHg increase	0.8 (0.7–1.02)	0.091	1.2 (0.9–1.6)	0.2
• SBP > 160 mmHg	0.6 (0.2–1.3)	0.2		
• SBP < 120 mmHg	3 (0.6–10)	0.2		
SBP changes during MT				
• Maximal SBP > 160 mmHg	0.7 (0.3–1.6)	0.7		
• Maximal SBP > 180 mmHg	0.5 (0.1–2.2)	0.3		
• Minimal SBP < 100 mmHg	2.1 (0.9–4.6)	0.07	4.7 (1.3–17)	0.019
• SBP decrease > 20%	0.8 (0.4–1.7)	0.5		
• SBP per 10% increase	2 (1.2–3.2)	0.004	1.9 (0.8–4.3)	0.13
• SD of SBP per 1-unit increase	1.01 (0.97–1.04)	0.5		
• CV of SBP per 1-unit increase	4.8 (1–300)	0.46		
Episodes of SBP exceeding the level of SBP observed before groin puncture:				
• Within a 2-h period after recanalization	3.6 (1.6–8.3)	0.002	5 (1.3–19)	0.021
• Immediately after recanalization	2.5 (1.2–5.4)	0.018		
• Within 2–24 h after MT	1.2 (0.9–1.6)	0.27		
SBP changes within 24 h after MT				
• Increase of SBP in comparison with SBP after recanalization >20%	2 (0.9–4)	0.06	3.4 (1.1–10)	0.035
• Decrease of SBP in comparison with SBP at the end of MT > 20%	0.78 (0.4–1.7)	0.5		
• Instances of SBP > 160 mmHg	1.3 (0.7–2.7)	0.4		
• Instances of SBP > 180 mmHg	1.2 (0.5–2.9)	0.7		
• Instances of SBP fall below 100 mmHg within 24 h after MT	1.9 (0.8–4.9)	0.2		
• Instances of SBP fall below 100 mmHg within 2–24 h after MT	3.4 (1.3–9)	0.019	3.2 (1.1–9)	0.039
• SD of SBP after MT per 1-unit increase	1.04 (0.99–1.096)	0.088	0.96 (0.9–1.04)	0.3
• CV of SBP after MT per 1-unit increase	1 (0.99–1.02)	0.18		

CI, confidence interval; CV, coefficient of variation; IQR, interquartile range; IV, intravenous; MT, mechanical thrombectomy; NIHSS, National Institutes of Health Stroke Scale; SBP, systolic blood pressure; SD, standard deviation.

The multivariate analysis of patients who developed UnND later than 24 h and who did not develop deterioration signs within 24 h revealed that SBP > 180 mmHg on admission, episodes of SBP exceeding the level of SBP observed before groin puncture and occurring within a 2-h period after recanalization and SBP drop <100 Hg during MT procedure (Supplemental Table 6) were significantly associated with higher likelihood of UnND developing later than 24 h but during a 7-day period. Owing to multicollinearity, SD was not included in the multivariate analysis.

## Discussion

There were 5.9% patients with UnND within 24 h and 8.3% patients with UnND within a 7-day period after MT among patients with successful recanalization. It comprises respectively 4.9% and 6.9% of all patients who underwent MT.

The relationship between SBP and UnND was evaluated in three different treatment periods: before MT, during MT, and during 24 h after MT. Classically, the relationship between SBP and clinical outcome is described as U/J shaped, with both high and low values of SBP being independent prognostic factors for a poor outcome.^20^ In our study, the relationship between SBP and UnND was dependent on treatment period. Only during 24-h period after MT for patients with successful recanalization, the J/U-shaped relationship between SBP and UnND was found while in other periods the hypothesis about J/U resembling relationship between SBP and UnND was our challenge which we were unable to evaluate. Whereas during 24 h after MT, the increase of SBP was a more pronounced prognostic factor for UnND in comparison with SBP decrease, that is, lower SBP was tolerated better than higher SBP despite exclusion of patients with early intracranial hemorrhage. It is notable that differences in study population (different recanalization status, including patients with sICH or not in the outcome group, etc.), methods of analysis, different outcome (early deterioration, mortality, functional dependence on 90 day, etc.) could affect the comparability of other studies to our results.

### SBP before MT

Our study found that despite the exclusion of patients with early intracranial hemorrhage, an elevated SBP on admission (>180 mmHg) was associated with UnND within 7 days. This is in agreement with the study of Girot *et al.*^2^ about the predictors of UnND. Meanwhile, there was no association between lower SBP observed on admission and UnND. In general, an elevated BP is common in stroke patients.^21^ Moreover, the relationship between high SBP and the outcome was observed independent of the heterogeinity of population in many studies. Otherwise, the association between low SBP and outcome differed and was more dependent on the study population and the outcome definition. A classical J/U-shaped relationship was established by Mulder *et al.*^22^ Maïer *et al.*^23^ found a J-shaped relationship for mortality, whereas a linear curve with a threshold at 180 mmHg was documented for a functional outcome what suggests potential differences in the pathophysiological etiology for clinical prognosis. Malhotra *et al.*^10^ found the association between high SBP and an unfavorable outcome in patients with successful recanalization and did not confirm the J/U-shaped relationship between the SBP and the outcome. Therefore, the pathophysiology of the hypertensive response in AIS is not completely understood,^21^ and the goal of the optimal antihypertensive treatment before MT is still under discussion.

### SBP during MT

The association between SBP during MT and UnND resembled J/U-shaped association although we were unable to confirm all the results in multivariate analysis. In univariate analysis, we found that SBP increase during MT in comparison with pre-procedural SBP is associated with UnND both during 24 h and 7-days although this association failed in multivariate analysis. Particularly, it may be due to a small number of patients with UnND and heterogeinity of population (different anesthesia type which is associated with the BP level during MT, etc.). Low SBP < 100 mmHg was associated with UnND within 7 days. This is in agreement with Maïer *et al.*^17^ that SBP drop during MT below 100 mmHg may be associated with a worse functional outcome. Petersen *et al.*^24^ demonstrated that larger intraprocedural BP reductions and sustained relative hypotension were both independently associated with a worsened functional outcome, regardless of the reperfusion grade. However, in later study by Maïer *et al.*,^25^ hypotension displayed a trend toward worse outcomes for patients with a poor collateral status only. Meta-analysis done by Malhorta *et al.*^10^ showed that increasing levels of maximum SBP were independently associated with lower odds of 3-month functional independence. However, authors Malhorta *et al.*^10^ were also unable to evaluate the hypothesis of a U-shaped relationship between the SBP parameters and the clinical outcomes of patients with AIS treated with MT. As in the case of pre-procedural studies, the hypothesis about the J/U-shaped relationship between SBP and the outcome and especially the impact of low SBP on the outcome is a matter of discussion.

### SBP within 24 h after MT

Our study demonstrated a direct association between SBP increase especially during the first 2 h after MT and UnND both within 24 h and 7 days. Data of many authors about SBP after MT has a complex association with outcomes, which differs mainly based on recanalization status. In Petersen *et al.*’s study,^24^ J/U-type association between SBP after MT and poor outcome was demonstrated. Our study does not contradict J/U-type association between SBP after MT and UnND. UnND was associated with low SBP (less than 100 mmHg), and SBP increase during 24 h after MT although we did not identify the optimal highest threshold after MT that may predict UnND. The increase level depended on SBP before MT and therefore should be considered individually. This is in agreement with the authors that any optimal threshold exists for post-MT BP after reperfusion status is unclear.^26^ It could be due to the fact that the same maximal SBP value might have a different effect on the stroke outcome depending on the hypertension history of individual patients and the type of anesthesia. Our study is also in agreement with other authors that the patients with successful recanalization during MT or those who underwent thrombolytic therapy tolerated lower SBP better and the favorable outcomes were shifted toward lower SBP values for patients with successful recanalization as compared to patients with unsuccessful recanalization.^20,24,25^ Moreover, our study is in agreement with Anadani *et al.*^9^ that the association between positive SBP increases and outcomes were more pronounced than the association between SBP reduction and outcomes. However, a drop of SBP below 100 mmHg, especially during the later period, that is, 2–24 h after MT was associated with UnND. Although the future studies are needed to specify the magnitude of the lowest SBP during all the treatment phases associated with UnND, our observations might support the hypothesis^23^ that low SBP (<100 mmHg) during MT and within 2–24 h after recanalization might lead to hypoperfusion of ischaemic penumbra and an increase in the infarction size.

It is notable that despite many studies, BP treatment before, during, and after MT is inconsistent and remains unclear whether BP changes is a cause or just a poor outcome marker.

Carvalho Dias *et al.*^18^ noticed that a spontaneous early BP drop after MT is a marker of successful reperfusion and therefore is a marker of improvement of cerebral autoregulation due to the reduced final ischemic core. Thus, this can explain our results that the absence of the decrease in SPB or even a slight rise observed immediately after MT in the first 2 h is related to UnND. There are reports that due to severe damaged microcirculation, namely, microvascular thrombosis, cerebral edema, and microemboli formation or in the presence of a few collaterals, reperfusion may be incomplete even in successful recanalization.^18,27^ The absence of SBP decline after MT is likely to be a marker of insufficient reperfusion and therefore is associated with penumbra/infarct growth beyond the initial penumbra what may lead to UnND.^28^ In addition, we found that SBP instability during 24 h after MT as expressed with the episodes of SBP increase of more than 20% compared with SBP after recanalization was associated with UnND occurrence within a 7-day period despite exclusion of patients with early intracranial hemorrhage. Anadani *et al.*^9^ found that the greater the SBP increase post-MT, the higher the risk for unfavorable outcome and ND after MT. And vice versa, Carvalho Dias *et al.*^18^ hypothesized that normalization of SBP within 24 h after recanalization may correlate with earlier reperfusion of ischemic penumbra. One of the possible mechanisms of the influence of such a significant SBP increase on UnND is that a cerebral autoregulation is impaired in patients with AIS after stroke and a larger increase of SBP during all 24 h period and not only for a short time after MT might directly affect the brain tissue, leading to the growth of the ischemic lesion despite successful recanalization. Otherwise, a larger SBP increase after MT may be caused by microcirculatory disturbances such as distal microembolization that can occur from the disrupted clots and partially block the collateral flow leading to ischemic lesion extension. The mechanism of small branch occlusion within the atherosclerotic plaque could not be excluded, either. On the contrary, without having the data about the perfusion sequences at follow-up imaging, we are unable to confirm the hypothesis that UnND was mainly due to infarction extension.

The following predictors – the episodes of SBP increase >20% within 24 h after MT, and high SBP on admission were significantly associated only with ND developing within a 7-day period after MT without a significant association between ND developed at early 24 h post-MT. It may be partly associated with both the increasing number of ND including UnND during a longer time period and the type of anesthesia applied. General anesthesia was applied to almost half of patients and might mask the stroke severity within 24 h after MT. Otherwise, the extension of penumbra/cerebral infarction may occur later than 24 h after MT what is consistent with the studies which analyzed ND from 24 h to 15 days.^6^

Several limitations of this study should be noted. First, this is an observational study. Second, the relatively few numbers of patients with UnND and too few SBP measurements were included in our database and the strategy for SBP control was not standardized. Third, we did not measure the potential influence of collateral blood supply which may partly explain the findings. Fourth, the perfusion data are not available within 24-h 7-day period after MT.

Despite the above-mentioned limitations, our results suggest that the relationship between SBP and UnND is dependent on the treatment period. The J/U-shaped relationship between SBP and UnND was found only in patients with successful recanalization during a 24-h period after MT, while the hypothesis about J/U relationship between SBP and UnND in other periods was not possible for us to evaluate. However, the increase of SBP during 24 h after MT was a more pronounced prognostic factor for UnND in comparison with the decrease in SBP.

To our mind, a further study with a prospective design investigating the causal relation between the SBP variability, especially following MT and UnND is needed. We think that more attention should be paid to the individualization of therapy. The factors for individualization of therapy might include recanalization status, stroke severity, BP before MT, and some additional variables. The studies with perfusion data and SBP measurements during different treatment phases may be helpful to better apprehend the SBP changes in the occurrence of UnND.

## Supplemental Material

sj-docx-1-tan-10.1177_17562864221093524 – Supplemental material for Association between systolic blood pressure parameters and unexplained early neurological deterioration (UnND) in acute ischemic stroke patients treated with mechanical thrombectomy Click here for additional data file. Supplemental material, sj-docx-1-tan-10.1177_17562864221093524 for Association between systolic blood pressure parameters and unexplained early neurological deterioration (UnND) in acute ischemic stroke patients treated with mechanical thrombectomy by Aleksandras Vilionskis, Virginija Gaigalaite, Lukas Salasevicius and Dalius Jatuzis in Therapeutic Advances in Neurological Disorders
