# Supplementary material for: Association between systolic blood pressure parameters and unexplained early neurological deterioration (UnND) in acute ischemic stroke patients treated with mechanical thrombectomy
Source: Ther Adv Neurol Disord. 2022 Jun 16;15:17562864221093524. doi: 10.1177/17562864221093524 (PMC9210098; doi:10.1177/17562864221093524)
Supplement: sj-docx-1-tan-10.1177_17562864221093524 – Supplemental material for Association between systolic blood pressure parameters and unexplained early neurological deterioration (UnND) in acute ischemic stroke patients treated with mechanical thrombectomy [file sj-docx-1-tan-10.1177_17562864221093524.docx]

Supplemental Table 1. Baseline characteristics divided by early neurological outcome after MT within 24 h.

|  | Overall without deterioration  N=358 | Early neurological improvement  N=250 | UnND  N=24 | P1-value | P2-value |
| --- | --- | --- | --- | --- | --- |
| Male, n (%) | 40.8 | 44 | 25 | 0.12 | 0.072 |
| Age, year, median (IQR) | 74 (65-80) | 72 (64-80) | 74 (70-77) | 0.54 | 0.69 |
| Atrial fibrillation, n (%) | 54.7 | 52.8) | 58.3 | 0.73 | 0.6 |
| Diabetes mellitus, n (%) | 12.8 | 13.6 | 16.7 | 0.59 | 0.67 |
| Hypertension, n (%) | 79.3 | 78.4 | 100 | 0.013 | 0.011 |
| Congestive heart failure, n (%) | 19 | 20.8 | 8.3 | 0.19 | 0.14 |
| Previous stroke, n (%) | 11.2 | 8.8 | 8.3 | 0.67 | 0.9 |
| Baseline NIHSS, median ( IQR) | 17 (13-20) | 16.5 (14-20) | 18 (12-21) | 0.59 | 0.41 |
| IV thrombolysis before thrombectomia, n (%) | 63.7) | 64.8 | 75 | 0.26 | 0.31 |
| TACI, n (%) | 41.9 | 37.6 | 41.7 | 0.9 | 0.7 |
| General anesthesia, n (%) | 43 | 40 | 50 | 0.5 | 0.34 |
| Time from the onset to recanalization, min, median (IQR) | 252 (205-307) | 230.5 (199-274) | 257 (201-309) | 0.85 | 0.46 |

***Abbreviations*. MT-mechanical thrombectomy; NIHSS-** National Institutes of Health Stroke Scale, UnND- unexplained neurological deterioration, IQR (interquartile range), IV=intravenous, TACI-total anterior circulation stroke, p1-comparison between UnND and patients without UnND, p2-comparison between UnND and early neurological improvement groups.

Supplemental Table 2. Hemodynamic parameters divided by early neurological outcome within 24 h.

| Hemodynamic parameters | Overall without neurological deterioration  N=358 | Early neurological improvement  N=250 | UnND  N=24 | P1-value | P2-value |
| --- | --- | --- | --- | --- | --- |
| SBP on admission, median (IQR) | 160 (140-180) | 155 (140-170) | 160 (144.5-177.5) | 0.75 | 0.4 |
| SBP before groin puncture , median (IQR) | 150 (135-166) | 150 (140-160) | 147.5 (130  -160) | 0.49 | 0.5 |
| SBP during MT |  |  |  |  |  |
| - Mean SBP ±SEM | 141.5±0.9 | 141.1±1.1 | 140.1±2.3 | 0.6 | 0.7 |
| - Lowest SBP before recanalization, median (IQR) | 125 (110-140) | 125 (110-140) | 115 (100-132.5) | 0.45 | 0.4 |
| - Highest SBP, median (IQR) | 150 (138-165) | 150 (140-160) | 160 (146-170) | 0.22 | 0.1 |
| - Difference between highest and lowest SBP during MT, median (IQR) | 20 (10-40) | 20 (10-35) | 32.5 (16.2-55) | 0.1 | 0.05 |
| SBP after recanalization, median ( IQR) | 140 (120-150) | 140 (120-150) | 127.5 (125-150) | 0.6 | 0.5 |
| SBP during 24 h after MT |  |  |  |  |  |
| - Mean SBP±SEM | 144.2±0.7 | 141.9±0.8 | 138.6±3 | 0.07 | 0.24 |
| - Highest SBP, median (IQR) | 163 (151-175) | 160 (150-170) | 165(150-170) | 0.8 | 0.5 |
| - Lowest SBP, median (IQR) | 130 (120-140) | 130 (120-140) | 125 (106-140) | 0.6 | 0.7 |

***Abbreviations*. MT-mechanical thrombectomy; NIHSS-** National Institutes of Health Stroke Scale, UnND- unexplained neurological deterioration, IQR (interquartile range), IV=intravenous, SBP-systolic blood pressure, SEM- standard error of mean, p1-comparison between UnND and patients without UnND, p2-comparison between UnND and early neurological improvement groups.

Supplemental Table 3. Logistic regression analysis of independent predictors of UnND within 24 h in patients with successful recanalication and no procedural and hemorrhage complications (only patients with early neurological improvement and UnND were included, n=274).

|  | Univariate analysis | | Multivariate analysis | |  |
| --- | --- | --- | --- | --- | --- |
|  | Odds ratio, 95% CI | p-value | Odds ratio, 95% CI | p-value |  |
| Male | 0.42 (0.2-1.1) | 0.07 | 0.5 (0.18-1.2) | 0.046 |  |
| Age per 10 year increase | 1.006 (0.7-1.4) | 0.9 |  |  |  |
| NIHSS score per 5 points increase | 1.2 (0.8-1.7) | 0.3 |  |  |  |
| Atrial fibrillation | 1.3 (0.5-2.9) | 0.6 |  |  |  |
| Diabetes mellitus | 1.2 (0.5-2.9) | 0.6 |  |  |  |
| Congestive heart failure | 0.4 (0.1-1.5) | 0.16 |  |  |  |
| Previous stroke | 0.9 (0.2-4.2) | 0.9 |  |  |  |
| Glucose level at admission>6 mmol/l per 1 mmol /l increase | 0.3 (0.1-0.7) | 0.2 |  |  |  |
| General anesthesia | 1.5 (0.6-3.2) | 0.3 |  |  |  |
| IV thrombolysis before MT | 1.6 (0.7-4.2) | 0.3 |  |  |  |
| Time from the onset to recanalization per 30 min increase | 1.05 (0.8-1.3) | 0.6 |  |  |  |
| SBP on admission >180 mmHg | 1.3 (0.4-4.2) | 0.6 |  |  |  |
| SBP before groin puncture |  |  |  |  |  |
| SBP per 10 mmHg increase | 0.8 (0.6-1.1) | 0.17 |  |  |  |
| SBP >160 mmHg | 0.8 (0.4-1.9) | 0.67 |  |  |  |
| SBP <120 mmHg | 2.44 (0.8-7.9) | 0.04 | 3 (0.6 -9) | 0.2 |  |
| SBP changes during MT: |  |  |  |  |  |
| - SBP decrease > 20 percent | 1.05 (0.4-2.5) | 0.9 |  |  |  |
| - SBP per 10 percent increase | 2.2 (1.3-3.8) | 0.007 | 1.6 (0.8-3.5) | 0.18 |  |
| - Maximal SBP>160 mmHg | 1.4 (0.6-3.5) | 0.43 |  |  |  |
| - Maximal SBP>180 mmHg | 1.08 (0.3-4.9) | 0.9 |  |  |  |
| - Minimal SBP <100 mmHg | 1.4 (0.5-3.7) | 0.48 |  |  |  |
| - SD of SBP per 1- unit increase | 1.024 (0.98-1.084) | 0.2 |  |  |  |
| - CV during per 1-unit increase | 11.6 (1-200) | 0.3 |  |  |  |
| Episodes of SBP exceeding the level of SBP observed before groin puncture and occurring after MT: |  |  |  |  |  |
| - within a two-hour period after recanalization | 2.8 (1.1-6.7) | 0.025 | 2.9 (1-6.2) | 0.014 |  |
| - immediately after recanalization | 3.5 (1.5-8) | 0.005 |  |  |  |
| - within 2h-24 h after MT | 1.7 (0.8-3.9) | 0.2 |  |  |  |
| SBP changes within 24 h after MT |  |  |  |  |  |
| - Increase of SBP in comparison with SBP after recanalization |  |  |  |  |  |
| - Increase of SBP >10 percent | 1.2 (0.8-1.6) | 0.33 |  |  |  |
| - Increase of SBP > 20 percent | 1.4 (0.6-3.3) | 0.3 |  |  |  |
| - Decrease of SBP in comparison with SBP at the end of MT > 20 percent | 1.4 (0.4-4.3) | 0.5 |  |  |  |
| - Instances of SBP>160 mmHg | 1.6 (0.7-3.6) | 0.3 |  |  |  |
| - Instances of SBP>180 mmHg | 0.8 (0.2-3.8) | 0.8 |  |  |  |
| - Instances of SBP fall below 100 mmHg within 24 h after MT | 0.6 (0.2-2.7) | 0.5 |  |  |  |
| - Instances of SBP fall below 100 mmHg within 2- 24 h after MT | 3.4 (1.1-11) | 0.047 | 1.9 (1.1-3.6) | 0.036 |  |
| - SD of SBP after MT per 1-unit increase | 1.022(0.9-1.084) | 0.4 |  |  |  |
| - CV of SBP after MT per 1-unit increase | 1.03 (0.9-1.016) | 0.5 |  |  |  |

***Abbreviations*. MT-mechanical thrombectomy, NIHSS-** National Institutes of Health Stroke Scale, SBP-systolic blood pressure, IV-intravenous, IQR -interquartile range, CV- coefficient of variation , SD-standard deviation, CI-confidence interval

Supplemental Table 4. Baseline characteristics divided by early neurological outcome during a 7-day period

|  | Overall without neurological deterioration  N=298 | UnND  N=34 | P-value |
| --- | --- | --- | --- |
| Male, n (%) | 39.6 | 41.2 | 0.8 |
| Age, year, median (IQR) | 74(65-80) | 76 (70-79) | 0.26 |
| Atrial fibrillation, n (%) | 55 | 58.8 | 0.67 |
| Diabetes mellitus, n (%) | 11.4 | 17,6 | 0.29 |
| Hypertension, n (%) | 79.9 | 100 | 0.004 |
| Congestive heart failure, n (%) | 19.5 | 29.4 | 0.17 |
| Previous stroke, n (%) | 10.7 | 11.8 | 0.8 |
| Baseline NIHSS , median (IQR) | 17 (13-20) | 16 (12-21) | 0.9 |
| IV thrombolysis before thrombectomia, n (%) | 62.4 | 76.5 | 0.11 |
| TACI, n (%) | 43 | 41.2 | 0.8 |
| General anesthesia, n (%) | 43.6 | 52.9 | 0.3 |
| Time from the onset to recanalization, min, median (IQR) | 245(201-301) | 257 (210-275)- | 0.8 |

***Abbreviations*. MT-mechanical thrombectomy; NIHSS-** National Institutes of Health Stroke Scale, UnND- unexplained neurological deterioration, IQR (interquartile range), IV=intravenous, TACI-total anterior circulation stroke

Supplemental Table 5. Hemodynamic parameters divided by early neurological outcome during a 7-day period

| Hemodynamic parameters | Overall without neurological deterioration  N=298 | UnND  N=34 | P-value |
| --- | --- | --- | --- |
| SBP at admission, median (IQR) | 160 (140-176.5) | 175 (153-186) | 0.006 |
| SBP before groin puncture, median (IQR) | 150 (140-165) | 145 (138-156) | 0.1 |
| SBP during MT |  |  |  |
| - Mean SBP ±SEM | 142.1±1 | 138.2±2.6 | 0.22 |
| - Lowest SBP before recanalization, median (IQR) | 125 (110-140) | 125 (100-136) | 0.5 |
| - Highest SBP before recanalization, median (IQR) | 155 (140-170) | 155 (143-162) | 0.9 |
| - Difference between highest and lowest SBP during MT, median (IQR) | 20 (10-35) | 32 (20-40) | 0.06 |
| SBP after recanalization, median (IQR) | 140 (120-150) | 130 (118-143) | 0.2 |
| SBP during 24 h after MT, median (IQR) |  |  |  |
| - Mean SBP after MT±SEM | 144.1±0.8 | 140.1±2.4 | 0.11 |
| - Highest SBP after MT, median (IQR) | 163 (151-175) | 168 (153-177) | 0.25 |
| - Lowest SBP after MT, median (IQR) | 124 (112-136) | 120 (110-130) | 0.2 |

***Abbreviations*. MT-mechanical thrombectomy; NIHSS-** National Institutes of Health Stroke Scale, UnND- unexplained neurological deterioration, IQR (interquartile range), IV=intravenous, SBP-systolic blood pressure, SEM- Standard error of mean

Supplemental Table 6. . Logistic regression analysis of independent predictors of UnND occurrence during a 7-day period in patients with successful recanalization and no procedural and hemorrhage complications (patients with deterioration signs within 24 h were not included) (N=358)

|  | Univariate analysis | | Multivariate analysis | |
| --- | --- | --- | --- | --- |
|  | Odds ratio, 95% CI | p-value | Odds ratio, 95% CI | p-value |
| Male | 2.5 (0.9-7.1) | 0.082 | 5 (1.3-19) | 0.021 |
| Age per 10 year increase | 1.4 (0.9-2.1) | 0.19 |  |  |
| Atrial fibrillation | 0.8 (0.3-2.2) | 0.6 |  |  |
| Diabetes mellitus | 2.6 (0.8-8.4) | 0.11 |  |  |
| NIHSS score per 5 points increase | 1.1 (0.8-1.5) | 0.5 |  |  |
| IV thrombolysis before thrombectomy | 1.8 (0.6-5.8) | 0.3 |  |  |
| General anesthesia | 2.2 (0.8-7.1) | 0.13 |  |  |
| Onset recanalization per 30 min increase | 0.95(0.8-1.2) | 0.6 |  |  |
| Glucose level >6 mmol/l per 1 mmol/l increase | 0.9 (0.8-1.19) | 0.6 |  |  |
| SBP on admission >180 mmHg | 6.7 (2.4-19) | 0.0001 | 10.3 (2.7-39) | 0.001 |
| SBP before groin puncture <120 mmHg | 5 (1.25-20) | 0.029 | 2 (0.1-20) | 0.6 |
| SBP changes during MT: |  |  |  |  |
| - SBP decrease > 20 percent | 0.6 (0.2-1.9) | 0.4 |  |  |
| - SBP per 10 percent increase | 1.6 (0.8-3.2) | 0.16 |  |  |
| - Maximal SBP>180 mmHg | 1.4 (0.5-3) | 0.24 |  |  |
| - Minimal SBP <100 mmHg | 3 (1-8) | 0.042 | 5.9 (1.1-34) | 0.035 |
| - SD of SBP per 1-unit increase | 0.98 (0.94-1.04) | 0.6 |  |  |
| - CV of SBP per 1-unit increase | 0.92 (0.1-10) | 0.9 |  |  |
| Episodes of SBP exceeding the level of SBP observed before groin puncture: |  |  |  |  |
| - within a two-hour period after recanalization | 7.9 (1.8-35) | 0.007 | 8.9 (1.7-47) | 0.01 |
| - immediately after recanalization | 1.7 (0.5-8) | 0.4 |  |  |
| - within 2h-24 h after MT | 2.6 (0.9-7.5) | 0.064 | 0.9 (0.8-1.2) | 0.5 |
| SBP changes within 24 h after MT |  |  |  |  |
| - Increase of SBP in comparison with SBP after recanalization >20 percent | 4.2 (1.3-13) | 0.014 | 3.3 (0.8-14) | 0.1 |
| - Decrease of SBP in comparison with SBP at the end of MT > 20 percent | 0.87 (0.3-2.6) | 0.8 |  |  |
| - Instances of SBP>160 mmHg | 1.3 (0.6-2.7) | 0.4 |  |  |
| - Instances of SBP>180 mmHg | 1.8 (0.6-5.8) | 0.3 |  |  |
| - Instances of SBP fall below 100 mmHg within 24 h after MT | 1.6 (0.7-5) | 0.34 |  |  |
| - Instances of SBP fall below 100 mmHg within 2-24 h after MT | 2.2(0.6-18) | 0.2 |  |  |
| - SD of SBP after MT per 1-unit increase | 1.1 (1-1.2) | 0.008 |  |  |
| - CV of SBP after MT per 1-unit increase | 1.006 (0.99-1.02) | 0.38 |  |  |

***Abbreviations*. MT-mechanical thrombectomy, NIHSS-** National Institutes of Health Stroke Scale, SBP-systolic blood pressure, IV-intravenous, IQR -interquartile range, CV- coefficient of variation , SD-standard deviation, CI-confidence interval
